# Burnout or Depression? Investigating Conceptual and Empirical Distinctions in a High-Stress Occupational Group

**DOI:** 10.3390/jcm14124036

**Published:** 2025-06-07

**Authors:** Łukasz Baka, Monika Prusik, Krzysztof Grala

**Affiliations:** 1 Institute of Psychology, The Maria Grzegorzewska University, 40 Szczęśliwicka Str., 02-353 Warsaw, Poland; kgrala@aps.edu.pl; 2 Faculty of Psychology, University of Warsaw, 5/7 Stawki Str., 00-183 Warsaw, Poland; m.prusik@uw.edu.pl

**Keywords:** job burnout, depression, police officers, job stress, confirmatory factor analyses

## Abstract

**Background/Objectives:** Despite 50 years of research, there is still no consensus on whether burnout and depression are distinct constructs within the domain of occupational psychopathology. The overarching aim of this study was to address the question of whether burnout and depression represent manifestations of the same phenomenon, based on data collected from a sample of police officers working in Central Europe. **Methods:** The participants included a representative group of Polish police officers. They were selected using a probabilistic (random) sampling method. Correlational analyses were conducted to examine the relationships between burnout (including the dimensions of exhaustion and disengagement) and depression. **Results:** The results indicated that although burnout and depression are closely related, they are not identical phenomena. This conclusion was further supported by the results of confirmatory factor analyses (CFAs), which demonstrated that the dimensions of disengagement and exhaustion did not overlap with the construct of depression. **Conclusions:** As psychiatrists have observed, individuals diagnosed with burnout often also meet criteria for mental disorders such as somatoform, affective, or anxiety disorders. As psychologists, we may continue to debate whether burnout and depression are conceptually equivalent; however, it remains equally important to provide support to police officers experiencing burnout and/or depression.

## 1. Background

Job burnout and depression are among the most frequently studied mental health consequences of job stress over the past fifty years, second only to PTSD [1]. For example, when combined with the term “job stress”, the EBSCOhost database (PsycINFO and PsycEXTRA; 19 October 2024) returns 11,381 records for burnout and 2355 records for depression. Both scientists and practitioners are interested in these phenomena due to their potentially severe negative consequences at both the individual (e.g., diabetes, cardiovascular disease [2,3]) and organizational levels (e.g., absenteeism, job turnover [4]).

Since the term “job burnout” was first introduced in the literature [5], there has been ongoing debate regarding the extent to which it constitutes a distinct theoretical construct or whether it overlaps with other, “genealogically older” phenomena in health psychology, such as depression. Additionally, scholars continue to discuss whether the symptoms associated with job burnout are unique and exclusive to this construct [4,6]. Despite five decades of research, it remains unclear whether job burnout should be considered a separate phenomenon or a job-related variant of depression [7,8]. Researchers [9,10,11] have pointed out that the majority of studies on this topic (a) have relied on the traditional three-factor model of job burnout, (b) have been conducted on relatively small and non-representative samples, and (c) have predominantly been carried out in Western Europe and North America. Therefore, there is a need for research based on models other than the three-factor model, conducted on representative and professionally homogeneous groups in culturally distinct regions.

In response to these expectations, this paper aims to explore whether job burnout—characterized by two components: exhaustion and disengagement from work [12]—and depression are manifestations of the same phenomenon or two distinct indicators of psychological ill-being. The analysis is based on data collected from a representative sample of nearly 9800 police officers working in Central Europe (Poland).

### 1.1. Conceptualization of Job Burnout

The understanding of job burnout has significantly evolved over the past fifty years [1]. Initially, following Freudenberger [5], it was considered a unidimensional phenomenon characterized by extreme exhaustion, originating from a mismatch between an individual’s expectations and the actual demands of the job, as well as their inability to achieve desired goals. Freudenberger’s [5] approach, along with subsequent job burnout models (e.g., refs. [13,14]), was conceptually expanded by Maslach and her colleagues [15,16]. Maslach et al. [15] conceptualized job burnout as a psychological syndrome composed of three dimensions: (a) emotional and/or physical exhaustion, (b) cynicism (a detached attitude toward people and work environment), and (c) reduced personal accomplishment or a lack of professional efficacy.

However, not all studies [17,18,19] support the three-factor structure of job burnout proposed by Maslach et al. [15]. Some researchers [19,20] suggest that the third component—inefficacy—develops independently and later than the other two dimensions, implying that it may be a consequence of burnout rather than a core symptom. In response to these findings, Demerouti et al. [12] proposed an alternative two-factor model of job burnout, comprising exhaustion and disengagement from work. According to their perspective [12], burnout occurs when high job demands (e.g., workload, emotional demands, role conflict) are not sufficiently counterbalanced by job and personal resources (e.g., autonomy, social support, self-efficacy). This prolonged imbalance results in energy depletion and emotional, physical, and cognitive exhaustion, as well as disengagement from work—a state characterized by a detached attitude toward clients, colleagues, and the broader work context, including job responsibilities, personal values, and organizational culture.

### 1.2. Conceptualization of (Clinical) Depression

According to the *Diagnostic and Statistical Manual of Mental Disorders*, 5th Edition, Text Revision (DSM-5-TR), major depressive disorder (MDD) is characterized by the presence of at least five of the following symptoms: (a) depressed mood, (b) diminished interest or pleasure in activities, (c) significant weight loss or gain, (d) insomnia or hypersomnia, (e) psychomotor agitation or retardation, (f) fatigue, (g) low self-esteem or excessive/inappropriate guilt, (h) difficulty concentrating, and (i) recurrent thoughts of death or suicide. Notably, these symptoms—particularly low mood and anhedonia—must persist for most of the day, nearly every day, for at least two weeks. Additionally, they must result in significant functional impairment and cannot be attributable to other causes, such as substance use, medication effects, or bereavement.

In the *International Classification of Diseases*, 11th Edition (ICD-11), depressive disorder (single episode or recurrent) is similarly defined by the presence of at least five out of ten specified symptoms, including (a) depressed mood, (b) diminished interest in activities, (c) difficulty concentrating, (d) low self-esteem or irrational guilt, (e) hopelessness about the future, (f) recurrent suicidal thoughts, (g) disrupted or excessive sleep, (h) significant changes in appetite or weight, (i) psychomotor agitation or sluggishness, and (j) decreased energy or fatigue. A depressive episode must persist for most of the day, nearly every day, for at least two weeks, with bipolar disorder explicitly excluded as a differential diagnosis.

From an etiological perspective, depression may be triggered by the loss of something significant to an individual (e.g., health, social status, or a loved one) or by exposure to chronic, unresolvable stress [21,22], including work-related stress [23,24] or resource depletion [25]. Additionally, dispositional factors such as negative affectivity (NA) and trait anxiety (TA) play a significant role in susceptibility to depression [2].

### 1.3. Distinction Between or Overlap of Job Burnout and Depression? Theoretical Debate

The question of whether burnout and depression are manifestations of the same phenomenon or two distinct indicators of ill-being remains a subject of ongoing debate [1]. Some researchers emphasize several common features between the two constructs, including similarities in etiology, symptoms, and course [9,26,27,28,29]. First, stress and the depletion of personal resources play a significant role in the development of both job burnout and depression [11,25]. Moreover, similar symptoms can manifest in both conditions, such as constant feelings of being overwhelmed, fatigue, lack of energy, helplessness, feelings of worthlessness, and low self-esteem [30], as well as physical health problems like diabetes and cardiovascular disease [2,3]. As early as 1974, Freudenberger [5] emphasized that a burned-out individual looks, acts, and seems depressed (p. 161). Similarly, Maslach and Leiter [31] noted that individuals with high job burnout not only demonstrate the presence of negative emotions more often than others, but they also experience an absence of positive ones (p. 28), explicitly linking two core symptoms of depression—dysphoria and anhedonia—with job burnout. More recently, Schonfeld and Bianchi [32] have advocated for abandoning the ambiguous construct of job burnout in favor of the more clinically grounded paradigm of occupational depression—a form of depression in which symptoms are directly attributable to adverse work conditions. Their conceptualization aligns with psychiatric diagnostic standards (DSM-5), encompassing the nine core symptoms of major depressive disorder. The proposed framework incorporates causal attribution to occupational stressors and introduces the Occupational Depression Inventory (ODI [32]) as a validated diagnostic tool. This instrument assesses depressive symptoms within the context of one’s job, frames them within the nosology of mood disorders, and facilitates systemic responses, including the allocation of health benefits and workplace interventions.

In contrast, proponents of the distinction between the two constructs [33] (e.g., ref. [33]) argue that “burnout and depression are separate entities, even though they may share several common characteristics” (p. 218). These researchers [4,15,34,35] contend that while job burnout contextually refers to the work environment, depression can apply to all aspects of life. According to Warr [35], depression may be perceived as context-free affective well-being, while job burnout specifically concerns job-related affective well-being. In various job burnout models, researchers unanimously emphasize that different job characteristics are the primary sources of burnout. They define it as (a) “a state of physical and emotional depletion resulting from work conditions” ([5], p. 159); (b) “a crisis in one’s relationship with work in general” ([36], p. 296); (c) “a special type of prolonged occupational stress, particularly arising from interpersonal demands at work” ([4], p. 8); (d) “a prolonged response to chronic emotional and interpersonal stressors at work” ([15], p. 397); and (e) “a consequence of exposure to chronic job stress” ([37], p. 626). Although stress can be a source of both job burnout and depression, cross-sectional and cross-lagged studies have shown that job burnout predictors include chronic job-related stressors [34,38], while acute stressors related to negative life events and traumas are primary sources of depression [39,40].

### 1.4. Distinction Between or Overlap of Job Burnout and Depression? A Review of the Evidence

The issue of the overlap or distinction between occupational burnout and depression has been the subject of several reviews and meta-analyses [8,41,42,43]. For example, Bianchi et al. [41] examined the overlap between burnout and depression across 14 samples of respondents (*N* = 12,417) from six countries (France, Finland, Switzerland, Sweden, Spain, and New Zealand), all employed in healthcare and education. The meta-analytic correlations revealed that (a) exhaustion (the core of burnout) is more closely associated with depressive symptoms than with other dimensions of burnout (i.e., detachment and efficacy) and (b) the relationship between exhaustion and depression is strong enough (*r* = 0.80) to suggest an overlap between the two constructs. This overlap between the core of job burnout (exhaustion) and depression was also suggested in the meta-analysis by the results of 14 exploratory structural equation modeling bifactor analyses [41].

However, not all meta-analyses show such a strong relationship between these two constructs. For example, Meier and Kim [8], synthesizing 69 studies with 196 burnout–depression correlations (*N* = 46,191), found an overall effect size of *r* = 0.55 between the emotional exhaustion subscale of the Maslach Burnout Inventory (MBI; [44]) and depression [*r* = 0.49 between total burnout and depression as measured by other instruments]. Chen and Meier [42], summarizing 37 studies on samples of nurses, identified an overall effect size of *r* = 0.49 for the correlation between exhaustion (as a subscale of the MBI) and depression [*r* = 0.40 for total burnout and depression measured by other tools]. This result aligns with the findings of Koutsimani et al. [43], who analyzed 67 studies and found an overall effect size of *r* = 0.51 between exhaustion (in the MBI) and depression [*r* = 0.52 for total burnout and depression as measured by other tools]. A slightly lower (meta-analytic) correlation of *r* = 0.39 between burnout and depression was obtained by Glandorf et al. [45], who selected 13 studies on samples of athletes for analysis.

Ryan et al. [46], reviewing 61 articles on the mental health of physicians, reported that the magnitudes of the correlation coefficients between burnout and depression in these studies ranged from *r* = 0.41 to *r* = 0.71. Although significant, these correlations do not provide a definitive answer to the question of whether occupational burnout and depression are the same phenomenon. Researchers [8,43] note that differences in the magnitude of the correlation between burnout and depression may be moderated by (a) respondent gender [female predominance—higher correlations]; (b) respondent age [older respondents—higher correlations]; (c) years of employment [longer employment—higher correlations]; (d) profession [e.g., Koutsimani et al. [43]: educational staff *r* = 0.68 vs. general employed population *r* = 0.49]; (e) number of items and reliability estimates of burnout measures [higher reliability of tools—higher correlations]; and (f) study quality [e.g., Koutsimani et al. [43]: studies of average quality *r* = 0.57 vs. studies of good quality *r* = 0.49].

Glandorf et al. [45], after reviewing 11 longitudinal studies (on samples of athletes), provide strong evidence that burnout may be a predictor of (a) depression, (b) insomnia, and (c) reduced life satisfaction. According to Koutsimani’s research team [43], it is possible that employees diagnosed with depressive and/or anxiety disorders also experience burnout. The simultaneous presence of anxiety, depression, and burnout can complicate accurate diagnosis (e.g., “it’s only depression”) and hinder the selection of appropriate treatments. Unfortunately, research by Glandorf et al. [45] also shows that the negative effects of burnout and depression can reinforce one another.

### 1.5. Occupational Stress and Its Consequences (Including Burnout and Depression) in Police Officers

The issue of occupational stress and its consequences for police officers deserves separate discussion.

Systematic literature reviews (e.g., refs. [47,48]) indicate that police officers mainly have to contend with so-called operational and organizational stress factors at work. The former include, for example, hatred and aggression from other people (including entire crowds), the need to use physical force, contact with victims of crime (e.g., rape victims), negative public opinion, and hoax calls [47]. On the other hand, the latter include role ambiguity/conflict, lack of support from superiors, conflicts in cooperation with colleagues, organizational injustice (including discrimination), bureaucracy, long working hours and shift work (generating a sense of overload), and subjectively low salary [47,48].

These factors are not without impact on the mental health of police officers. For example, a meta-analysis of 13 research articles (1149 studies; 3722 police officers) by [48]) indicates that the overall prevalence of poor sleep quality in police officers is 51%. Subsequently, a meta-analysis of nine studies by Rostami et al. [49] indicates that one in four police officers (26.2%) suffer from metabolic syndrome (closely linked to type 2 diabetes and cardiovascular disease). Similarly, a systematic review (44,172 police officers) by Sousa et al. [50] reports that one in four police officers (26%) struggle with depression. A study by Queirós et al. [51] of a sample of 2057 police officers from the National Portuguese Police (a force policing urban centers) indicates that 85% of respondents presented high levels of operational stress; 11% exhibited symptoms of job burnout; and 55% were at risk for psychiatric disorders. A meta-analysis by Syed et al. [52] (60 cross-sectional and 7 longitudinal studies; 272,463 police personnel from 24 countries) shows that 14.6% of police personnel struggle with depression; 14.2% with PTSD; 9.6% with GAD; 8.5% with suicidal thoughts; 5.0% with alcohol dependence; and 25.7% with hazardous drinking.

Syed et al. [52] also reported that the strongest risk factor for depression and suicidal thoughts in police officers is high occupational stress, and the strongest risk factor for PTSD is high occupational stress and avoidant coping strategies. Also, a review of 29 studies by Galanis et al. [47] indicated that negative and maladaptive coping strategies (e.g., self-distraction, denial, self-blame, lack of control, and avoidance) exacerbate work-related stress. Importantly, a factor that reduces the risk of PTSD in police personnel is peer-support [52].

Occupational stress is also a correlate of burnout (in police officers)—in a meta-analysis of 39 studies (14,089 Chinese police officers) by Zhou et al. [53], the meta-analytic correlation between work pressure and occupational burnout was positive and moderate (*r* = 0.41).

Systematic reviews [47,54] also show that, among police officers, factors such as (a) childhood trauma experiences, (b) “negative” personality traits: neuroticism (anxiety, worry, mood disorders, and unpleasant feelings), psychoticism (inappropriate emotional expression), introversion, reduced resilience, and (c) “unhealthy” habits: reduced physical activity, lack of hobbies, and smoking—represent a risk for the emergence of various forms of psychopathology.

To the best of our knowledge, there is not yet a meta-analysis that presents the relationship between depression and burnout exclusively in groups of police officers. However, a meta-analysis of 10 studies (4572 police officers worldwide) by Ugwu and Idemudia [55] reports a positive and moderate meta-analytic correlation between burnout and PTSD among police officers (effect size = 0.34; this suggests no overlap between the two disorders). Additionally, a meta-analysis of 57 studies (6670 participants) conducted by Rytwinski et al. [56] shows that up to half (52%) of individuals with current PTSD may have co-occurring depression (MDD). Thus, the non-overlap between burnout and PTSD in police officers [55] may indicate that burnout and depression will not overlap in this occupational group either. Another example, in the cross-sectional study by Pikoulas et al. [57] on a sample of 396 Greek police officers, the relationship between emotional exhaustion (burnout core) and depression was found to be moderate (*r* = 0.54), thus not indicating any overlap between the two constructs.

### 1.6. Contribution of the Presented Study

Several aspects of this study warrant emphasis. First, sample size—this study was conducted on a randomly selected sample of 9793 respondents. This is particularly noteworthy, as many studies on job burnout have relied on non-representative samples [58]. For instance, in a review by Bianchi et al. [10], 34 out of 67 cross-sectional studies (51%) had fewer than 300 participants, while only 15 studies (22%) included more than 1000. Second, study location—our research was conducted in Poland (Central Europe), rather than in Western Europe or North America, where most previous studies have been conducted [10]. Third, the professional group examined—our study focused on police officers, a uniformed service. In contrast, most burnout research has been conducted on professionals in education and healthcare, with fewer studies analyzing mixed occupational groups [41]. Some reports suggest that the least resilient professions are in healthcare, healthcare support, and education [59]. For example, the global prevalence of depression is estimated at 34% among healthcare workers [60], 30.7% among teachers [61], and 14.6% among police personnel (8.5% for suicidal ideation, 9.6% for generalized anxiety disorder, and 14.2% for PTSD) [52]. Given that nurses and teachers experience particularly high levels of burnout and depression [59], distinguishing between these conditions in these professions can be challenging [62]. Fourth, gender distribution—our sample was predominantly male (83%), whereas most burnout research has been conducted on predominantly female samples [8]. Notably, women tend to report slightly higher levels of depression (*d* = 0.27; [63]). Finally, research methodology—we employed the two-factor Oldenburg Burnout Inventory (OLBI; [64,65]). In contrast, Bianchi et al. [10] reported that 80% of burnout studies have used the three-factor Maslach Burnout Inventory (MBI), which may introduce mono-operation bias and affect the validity of conclusions drawn [8].

## 2. Method

### 2.1. Instruments

*Job Burnout* was measured with the The Oldenburg Burnout Inventory (OLBI; [12]) in Polish version [65]. The questionnaire consists of 16 items assessing two burnout dimensions: exhaustion (a result of intense physical, affective, and cognitive strain) and disengagement (distancing oneself from work). Each subscale contains eight items, and responses are given on a 4-point Likert scale (1 = *strongly agree*, 4 = *strongly disagree*). A study by Demerouti et al. [66] demonstrated that the OLBI has satisfactory internal consistency (α = 0.73 for exhaustion; α = 0.83 for disengagement), a confirmed two-factor structure, and established convergent and discriminant validity.

*Depression* was measured with The Center for Epidemiological Studies-Depression Scale (CES-D [67]), in Polish version [68]. It is a brief self-report tool designed to measure depressive symptoms (e.g., sadness, anhedonia, fatigue, restless sleep, poor appetite) in the general population. The CES-D consists of 20 items assessing symptoms experienced over the past week, rated on a 5-point Likert scale (0 = *rarely or none of the time*, 4 = *most or almost all the time*). Scores range from 0 to 60, with higher scores indicating greater depressive symptomatology. Radloff [67] demonstrated that the CES-D has good internal consistency (α = 0.85–0.90), satisfactory test–retest reliability (*r* = 0.45–0.70), strong concurrent validity (based on clinical and self-report criteria), and established construct validity.

### 2.2. Participants

This study was conducted on a sample of 9793 Polish police officers, comprising 7907 men (83%) and 1680 women (17%). The age distribution within the sample was as follows: 3.5% were under 25 years old, 20.7% were between 25 and 30, 48.9% were aged 31 to 40, 22.2% were between 41 and 50, and 2.3% were over 50. The age categories were unevenly distributed, with an overrepresentation of officers aged 31–40 and an underrepresentation of those younger than 25 and older than 50, χ^2^(4) = 7135.53, *p* < 0.001.

The sample exhibited a high level of education, with 54.4% holding an academic degree. Additionally, 3.3% had post-secondary education, 37.4% had completed high school, and 0.2% had only basic or vocational education. The educational distribution showed an overrepresentation of individuals with university and secondary education and an underrepresentation of those with post-secondary or basic/vocational education, χ^2^(3) = 8600.189, *p* < 0.001. However, the prevalence of officers aged 31–40 and those with higher education is expected, as these characteristics are typical in this profession. Therefore, while age and education categories were not evenly distributed, they closely reflect the demographic structure commonly observed among Polish police officers.

Regarding professional experience, 12.3% of participants had less than three years of service, 35% had between three and ten years, 19.3% had 11–15 years, 14.4% had 16–20 years, 14.3% had 21–30 years, and 0.9% had over 30 years of service. A minority of respondents (13.9%) held managerial positions, while the majority (81.6%) worked in lower-ranking roles.

### 2.3. Procedure

Participants were selected using a probabilistic (random) sampling method. The study sample was drawn from the entire population of 97,462 Polish police officers through a stratified random sampling approach without replacement. The allocation process considered several factors, including region (voivodeship (voivodeship is the highest-level administrative subdivision of Poland, corresponding to a “province“ in many other countries; there are sixteen voivodeships in Poland), gender, job seniority, unit size, nature of duties, and type of service. A three-stage stratified sampling system was employed, incorporating segmentation. In the first stage, voivodeships were selected; in the second stage, police units were chosen based on their size; and in the third stage, officers were selected according to their professional profiles, which included gender, job seniority, and the type of service they performed. The proportional stratified sampling design ensured that the study sample was representative in terms of regional distribution, unit size, gender, job seniority, and service type.

Participants received a paper version of the questionnaire, accompanied by a letter explaining this study’s purpose. Full confidentiality and anonymity were guaranteed. Completed questionnaires were placed in envelopes and collected by research assistants, who were police psychologists. This study was conducted in compliance with the ethical principles outlined in the Helsinki Declaration. Of the 11,000 distributed questionnaires, 9793 (89%) were at least 85% complete and were therefore included in the data analysis. Prior to the main analysis, data mining procedures were applied. The sample structure and the sampling process were systematically controlled in accordance with the METADATA and DATA ENTRY protocols.

## 3. Results

The analysis started with the inspection of the missing data and check of the assumptions required to conduct Confirmatory Factor Analysis (CFA). CFA is a widely used statistical technique which allows examining the latent structure behind theoretical concepts, usually tested in a form of structural equation modeling (e.g., refs. [69,70,71]). The missing data did not exceed 1.25% for all data points (16 OLBI questions multiplied by the number of participants—156,688). The most prevalent pattern of missing data was related to not answering any of the 16 OLBI questions. We decided to exclude 95 participants (around 0.09% of the missing data) who did not answer at least 8 questions out of 16. For the remaining missing data (0.35%), we used the Expectation-Maximization (EM) algorithm for data replacement, as Confirmatory Factor Analysis (CFA) requires all missing data to be addressed to utilize its full functionality. Thus, the final sample with missing data replacement for OLBI was *N* = 9698. In case of CES, there was 1.43% of missing data (out of 195,860 data points). Participants who did not answer at least 10 questions out of 20 were excluded from further analyses. The remaining missing data were replaced using the EM algorithm. The final sample for CES included *N* = 9686 participants. The ultimate sample for OLBI and CES together consisted of 9671 people. The data were also screened in terms of other important assumptions (normal distribution but bearing in mind that our sample size overcomes the normality problem anyway due to the sample size, linear relationship between variables checked on randomly chosen pairs, as well as univariate and multivariate outliers).

### 3.1. The Factorial Validity of Burnout and Depression

**Figure A1:**
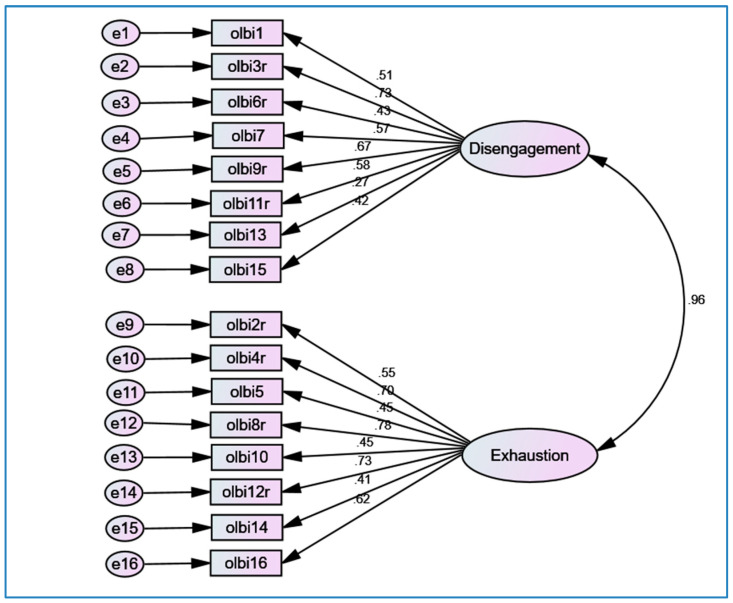
Confirmatory Factor Analysis for burnout—basic model (*N* = 9698).

**Figure A2:**
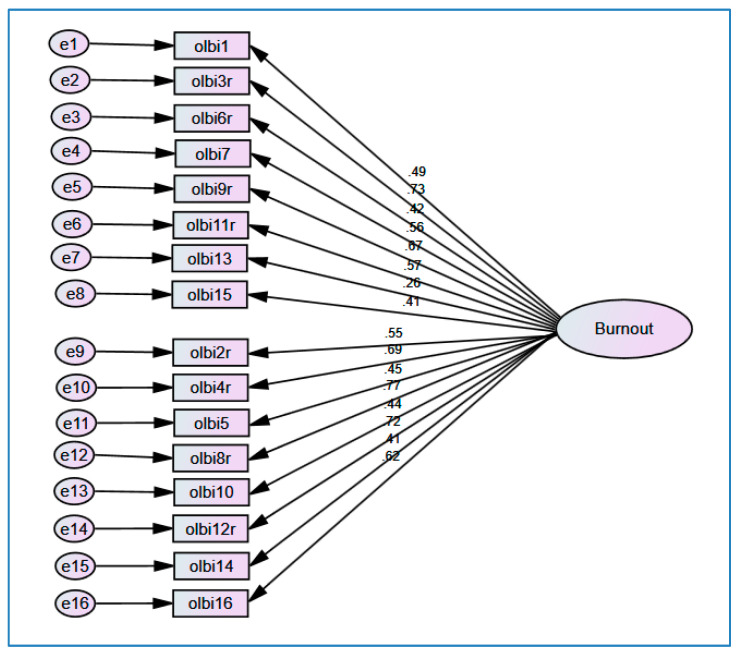
Confirmatory Factor Analysis for burnout—basic model with one—factorial solution (*N* = 9698).

As a first step, we decided to examine the factorial validity of burnout (the correlated two-factorial model) and depression (the one-factorial model). The Confirmatory Factory Analysis was preceded with analysis of basic descriptive statistics (Table 1). The results of CFA analysis for both constructs (burnout and depression) are presented in Table 2 and in Figure A1 and Figure A2 in the Appendix A.

**Figure A3:**
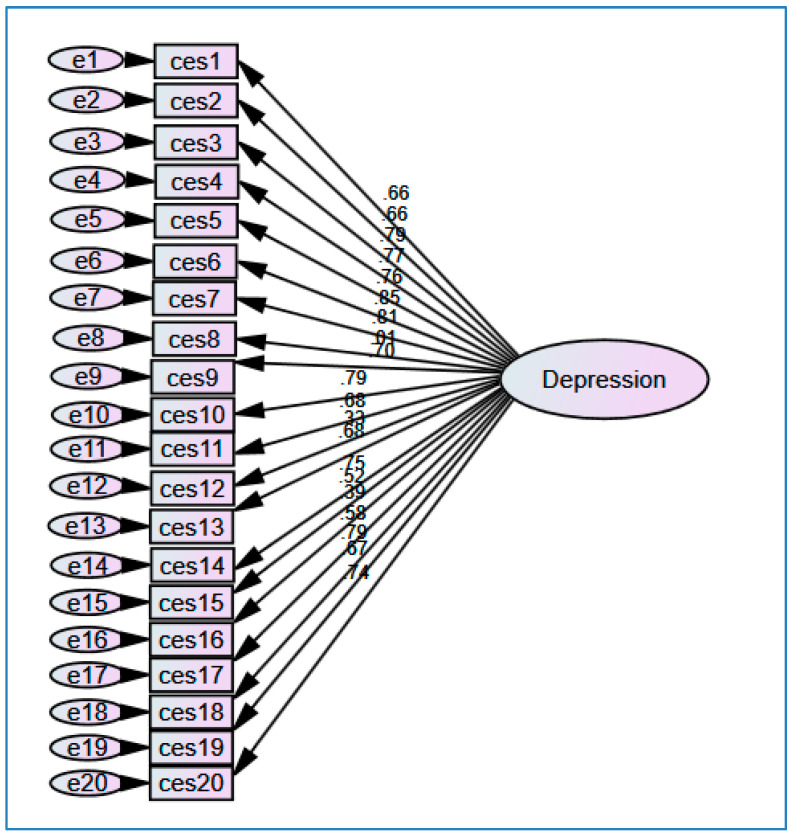
Confirmatory Factor Analysis for depression—basic model (*N* = 9698).

According to the results of the first two examined models (burnout, two-factorial solution, basic model, and a model with some respecifications added), the satisfactionary solution was not reached. Thus, two-factorial structure was not confirmed. Both models presented significant χ^2^ values, which could be considered as an indicator of a poor fit. However, in practice, significant χ^2^ test is very rarely used as an indicator of fit, since it is very difficult to obtain non-significant values [72,73], especially for large samples as in this case. Other, more conclusive, fit indices also suggested that the fit was not sufficient to accept the two-factor solution proposed by the scale’s authors, at least in the examined sample of Polish police officers. The ratio of χ^2^ to degrees of freedom was much above the desired interval of two and three [74] for both models. The root mean square error of approximation (RMSEA) was above the level of acceptance for the basic model and at the edge of acceptance for the model with respecifications (criteria of 0.06–0.08, or at least below 0.10). Also, the corresponding *p* of Close Fit (PCLOSE) for both models was less than the expected 0.05 for each model. The incremental fit index (IFI) was much lower than the recommended 0.95, and the standardized root mean square residual (SRMR) was above the recommended cutoff point (< 0.06). Akaike information criterion (AIC) values showed that the adjustments improved the basic model but still not to the point of acceptable results. In general, the improvement of the model with the estimated error covariances compared to the basic model was statistically significant, Δχ^2^(10) = 5378.12, *p* < 0.001. For both models, all indicators were statistically significant and ranging from 0.27 to 0.78 (for the standardized version) for the basic model and from 0.20 to 0.80 for the model with respecifications added. Thus, some of the regression weights were too low. The most problematic issue, however, was the value of correlation coefficients between “engagement” and “exhaustion” exceeding the commonly accepted value of *r* = 0.85 [75] and, in this way, suggesting serious discriminative validity issues. The value of correlation coefficient for the basic burnout model was *r* = 0.96, and for the burnout model with respecifications, it was *r* = 0.99. The additional analysis of Cronbach’s alpha revealed satisfactory levels of inter-reliability for both factors (α = 0.76 for disengagement and α = 0.81 for exhaustion). Overall, we could not accept the two-factor solution and proceeded to test a single-factor solution as a potentially better measurement of burnout (Table 2 and Figure A3 in the Appendix A). Against expectations, the one-factorial solution for burnout (basic model and a model with respecifications) had only slightly better performance. The χ^2^ test was significant and all the other indices suggested hardly acceptable fit. The ratio of χ^2^ to degrees of freedom was still not acceptable, but much better for the model with respecifications. IFI was slightly below the recommended value of 0.95 but only for the model with respecifications. SRMR and RMSEA were acceptable but also only for the model with adjustments. AIC value dropped significantly for the second model, which suggested an improvement in model fit. Again, all indicators were significant and ranging from 0.26 to 0.77 for the basic one-factorial model and from 0.22 to 0.77 for the model with respecifications. The respecifications based on error covariances of the second model significantly improved the one-factorial model for burnout, Δχ^2^(18) = 8242.36, *p* < 0.001. The Cronbach’s alpha for the one-factorial solution was α = 0.88.

We were not confident with the results (we could not accept any of the models with full confidence based on the values of fit indices) and decided to use Exploratory Factory Analysis (EFA) to check what a more adequate structure of burnout in the study sample would be. The detailed results of EFA are included in Table A1 in Appendix A. Based on the Cattell scree test and parallel test results, we examined several factorial solutions, including two to three factors using the PCA method in the initial runs of the procedure. An initial run of PCA was also used to determine the data factorability, which turned out to be good. The Kaiser–Meyer–Olkin measure of sampling adequacy was 0.92 (as recommended above 0.60), and the Bartlett’s test of sphericity was significant, χ^2^(120) = 54,080.92, *p* < 0.001. The diagonal values of the anti-image matrix were all above 0.50. We eventually settled upon the solution of two factors (allied with parallel test results). In the final run of the procedure, the Principal Axis Factoring (PAF) method of extraction was used together with Promax rotation. We used Promax rotation since the correlation coefficients for the considered dimensions were substantially correlated and did exceed 0.32 in the oblique solution [71]. The EFA indicated two factors which were centered on the direction of OLBI statements; reversed statements created one factor and non-reversed statements fell into the second factor. Apparently, the problem with fit in CFA analysis was due to problematic statistical performance of negatively worded items. This is a common problem and it was already discussed in the literature on many occasions [76].

Some argue that the disadvantages over benefits of using reversed statements are so large that negatively worded statements should not be used, at least in some language versions of psychological scales. Upon EFA results, we decided to relax requirements of previous CFA analysis and accept the one-factorial model which had values of fit indices at the edge of acceptable levels, but in light of the problematic performance of negatively reversed statements, we believe that the strict constraints of CFA should be slightly relaxed.

Next, we tested the structure validity of the depression scale (one-factorial solution as proposed by the authors). The outcomes of the CFA analysis are presented in Table 2 and in Figure A3 in Appendix A. The χ^2^ was significant for both models (basic one and the one with respecifications). The RMSEA value was acceptable but not great for the basic model, and it was very good for the model with respecifications. Unfortunately, *p* of Close Fit (PCLOSE) for both models was less than the expected 0.05 for each model. IFI was too low for the basic model and good for the model with respecifications. SRMR had acceptable values for both models. AIC dropped in value, which sugests that the second model had a better fit. The standardized regression weights were all significant except one (for the statement no. 8) and ranged between 0.01 (for the statement no. 8) and 0.85 for the simple model and 0.00 (statement no. 8) and 0.85 for the model with respecifications. The respecifications based on error covariances significantly improved the model for depression, Δχ^2^(9) = 7851.68, *p* < 0.001. Cronbach’s alpha was at the level of 0.93 for the depression scale. Overall, taking into acount all fit indices and respecifications, we decided to accept the one-factorial solution.

### 3.2. Internal Validity of Burnout and Depression

**Figure A4:**
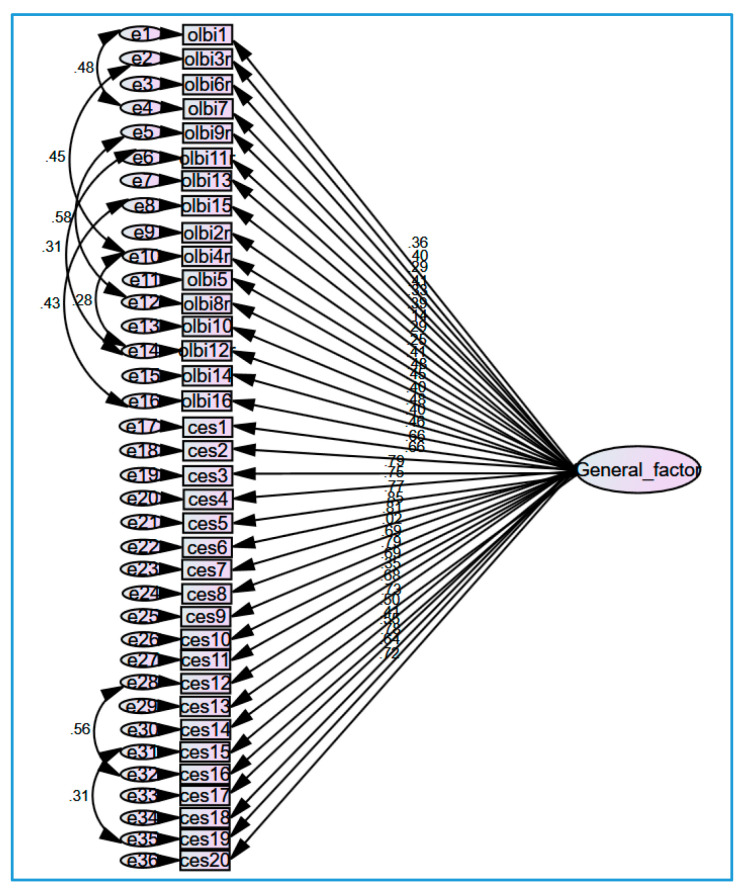
Confirmatory Factor Analysis for Model 1 with respecifications based on error covariances (*N* = 9671).

**Figure A5:**
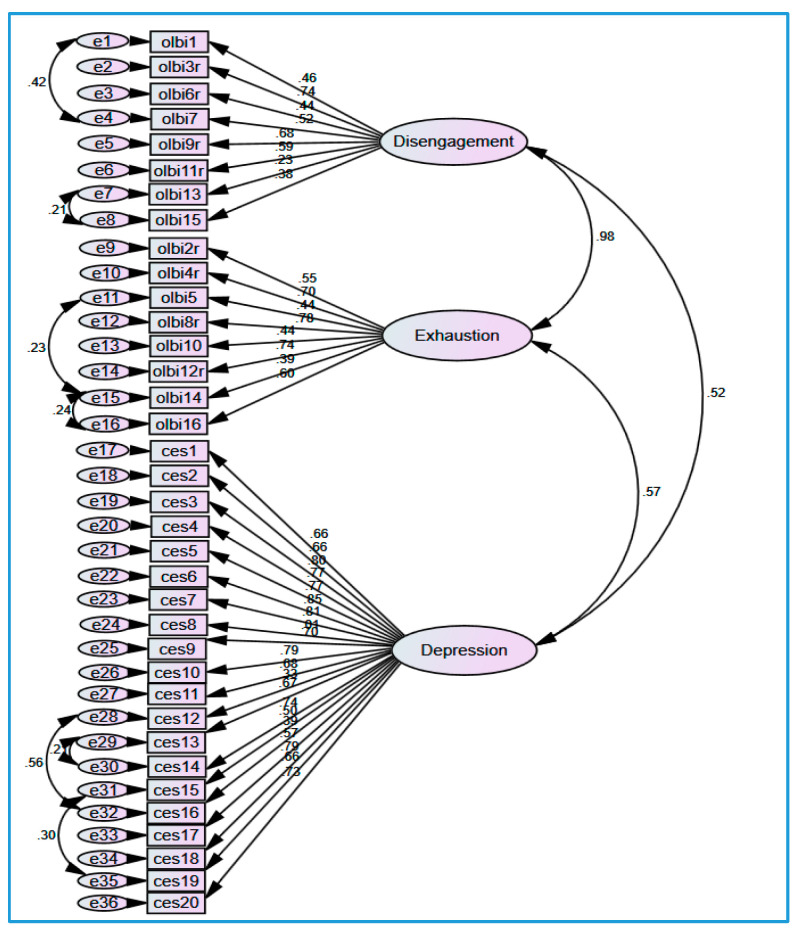
Confirmatory Factor Analysis for Model 2 with respecifications based on error covariances (*N* = 9671).

**Figure A6:**
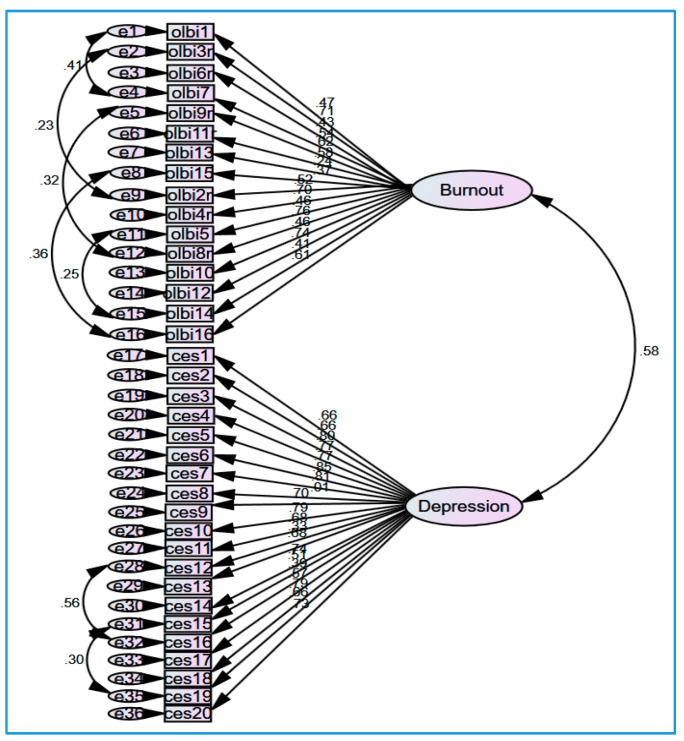
Confirmatory Factor Analysis for Model 3 with respecifications based on error covariances (*N* = 9671).

The previously tested models were combined into a common model in different configurations in order to test their mutual associations and to test if they possibly constitute subscales of one construct. Three models were tested. The first model (M1) assumes that disengagement, exhaustion, and depression are part of one common general depression factor. The second model (M2) takes into consideration that burnout originally consists of two subscales (disengagement and exhaustion). The third model (M3) examines the possibility that two subscales of disengagement and exhaustion should be treated as one general factor of burnout next to the depression factor. The results of CFA are presented in Table 3 and in Figure A4, Figure A5 and Figure A6 in Appendix A.

According to the M1 and M1 with respecifications, the adequate fit was not achieved. Respecifications based on error covariances did improve the fit of M1, Δχ^2^(8) = 17,858.01, *p* < 0.001, but other fit indices were still not acceptable. IFI and SRMR had no acceptable values for both models (M1 and M1 with respecifications). RMSEA had barely acceptable values, but PCLOSE was still below 0.05. AIC values dropped, which suggests an improvement after the addition of respecifications. The values of regression weights were all significant but some of them were very low since they ranged from 0.02 to 0.85 for M1 with respecifications and from 0.04 to 0.84 for M1 without respecifications. Cronbach’s alpha was very high (α = 0.94), but it does not mean that the M1 is really unidimensional.

Model 2 had a better performance in comparison to Model 1 based on the chi square test, Δχ^2^(3) = 22,905.62, *p* < 0.001, as well other indices of fit. RMSEA had better and acceptable values, IFI was better but still not great and below the level of acceptance. SRMR was slightly above the value of 0.06. The ratio of chi square to degrees of freedom also improved. All regression weights, except one for item no. 8 of the depression scale, were significant, and they ranged between 0.01 and 0.85. Respecifications of Model 2 had a significant effect, Δχ^2^(7) = 8144.33, *p* < 0.001, and they improved model parameters. IFI value improved but was still quite low; RMSEA had an acceptable level. The regression weights (except no. 8 for depression) were statistically significant and ranged from 0.001 to 0.85. The additional analysis of Cronbach’s alpha revealed good or very good levels of inter-reliability for both factors (α = 0.76 for disengagement and α = 0.81 for exhaustion), and α = 0.93 for the depression scale.

The fit of Model 3 was significantly better in comparison to that of Model 1, Δχ^2^(1) = 22,727.31, *p* < 0.001, but not of Model 2, Δχ^2^(2) = −178.31, *p* < 0.001. The fit of Model 2 was better than the fit of Model 3. The respecifications of Model 3 based on error covariances significantly improved the model fit, Δχ^2^(7) = 94,419.89, *p* < 0.001. Also, AIC values dropped. Other fit indices had very similar values as those related to Model 2. IFI was still not great and SRMR was a bit too high. The ratio of chi square to degrees of freedom was still not really acceptable, but this index is highly dependent on the sample size. RMSEA had good values for both: the basic model and the model with respecifications. The regression weights, again except one for item no. 8 of the depression scale, were significant and between 0.01 and 0.85 for the basic Model 3 and from 0.01 to 0.85 for the Model 3 with respecifications.

Overall, Model 2 with respecifications demonstrated the best fit among the tested models. Additional measures of composite reliability (CR; CR = 0.76 for disengagement; CR = 0.82 for exhaustion; CR = 0.93 for depression) and average variance extracted (AVE; AVE = 0.30 for disengagement; AVE = 0.37 for exhaustion; AVE = 0.43 for depression), together with previously reported Cronbach’s alpha measures (all above 0.70) and significant factor loadings, indicated acceptable convergent validity of Model 2, even though low AVE values still suggested some problems in the model.

In terms of discriminant validity, which was judged in our models based on correlations between factors as well as χ^2^ difference tests for competing models, we also inspected the ratio between squared AVE values and the constructs’ correlations, commonly known as the Fornell–Larcker criterion. The analysis revealed that depression was sufficiently distinguished from both disengagement and exhaustion (ratios: 0.67/0.52 for disengagement, and 0.67/0.57 for exhaustion), while disengagement was separate from depression (0.55/0.52) but not from exhaustion (0.55/0.98), and exhaustion was separate from depression (0.61/0.57), but not from disengagement (0.61/0.99). These findings provide additional support for the claim that depression and burnout are separate constructs, while there is substantial overlap between disengagement and exhaustion.

Bearing in mind the overall picture based on the different measures we employed, our findings suggest that burnout should be treated as two separate subscales (disengagement and exhaustion), even though the statistical parameters are not ideal and the two subscales are highly correlated. The results also demonstrate that depression is a fairly distinct construct and the claim that depression and burnout are part of the same construct is not supported by our data.

## 4. Discussion

As Meier and Kim ([8], p. 1) note “despite 35 years of study, burnout researchers have failed to reach a consensus about whether burnout is distinct from depression”. In other words, the debate over whether (job) burnout and depression are the same thing is still ongoing. The aim of this paper was an attempt to answer the question whether job burnout and depression are manifestations of the same phenomena or two distinct indicators of ill-being, based on data collected on a group of nearly 9800 police officers working in central Europe (in Poland).

The results of our analyses showed that the correlation between depression and generalized burnout was *r* = 0.56 (*p* < 0.01); between depression and exhaustion (the heart of burnout)—identically *r* = 0.56 (*p* < 0.01); and between depression and disengagement (the second factor of burnout)—slightly less, *r* = 0.47 (*p* < 0.01). Our results are analogous to those obtained in metanalysis: Koutsimani et al. [43] and Meier and Kim [8]. For example: in our study, the correlation between exhaustion (the core of burnout) and depression was *r* = 0.56; in Koutsimani’s team [43] study, *r* = 0.51; and in Meier and Kim’s [8] study, *r* = 0.55. In our study, the correlation between total burnout and depression was *r* = 0.56; in Koutsimani’s team [43] study, *r* = 0.52; and in Meier and Kim’s [8] study—slightly less, *r* = 0.49. Our findings are also consistent with recent research conducted by Roach et al. [77]. They found that symptoms of burnout—particularly emotional exhaustion—substantially overlap with symptoms of depression and anxiety. Nevertheless, statistical analyses suggest that burnout and depression remain distinct psychological constructs, indicating that they are not entirely synonymous.

Our study, like those conducted by other researchers [8,42,43], indicates that burnout and depression, although closely related, are not the same phenomenon. This was also supported by the results of our confirmatory factor analyses (CFAs), in which disengagement and exhaustion did not overlap with the depression construct. Thus, in the case of our study, the creation of a common top factor involving occupational burnout and depression proved unwarranted. Thus, in the case of our study, the creation of a higher-order factor including burnout and depression (a common factor) proved unwarranted.

This study has several notable strengths: (a) a large, representative sample (*N* = 9797); (b) a geographical context rarely studied—Poland, located in Central Europe, as opposed to North America or Western Europe; (c) an occupational group less frequently included in burnout research—police officers; (d) a predominantly male sample, which is underrepresented in many burnout studies; and (e) the use of the two-dimensional Oldenburg Burnout Inventory (OLBI; refs. [64,65]), a tool less commonly applied in comparative studies. At the same time, these strengths may also represent limitations, particularly in terms of sample diversity. Nevertheless, we believe this study helps to address several gaps identified in earlier research (e.g., refs. [8,10,41,58]).

This study employed a cross-sectional design, as our objective was to investigate the concurrent relationship between burnout and depression. Longitudinal research (e.g., ref. [45]) suggests that burnout may precede and predict the onset of depression, as well as other outcomes such as insomnia and reduced life satisfaction. However, our focus was not on determining temporal precedence. In general, as reported by Maske et al. [62], a diagnosis of occupational burnout is often comorbid with somatoform, affective, and anxiety disorders.

## 5. Conclusions

As noted by Maske et al. [62], individuals diagnosed with burnout often present with clinically significant mental disorders. The psychological community continues to debate whether burnout and depression are manifestations of the same condition [41], or whether depression emerges as a secondary consequence of burnout [45]. Regardless of the position one takes, it is essential to consider how best to support individuals experiencing job-related exhaustion. Meta-analyses [78,79,80,81] suggest that various interventions—including individual coaching, cognitive-behavioral therapy (CBT), rational emotive behavior therapy (REBT), relaxation techniques, and mindfulness-based programs—may be effective in reducing burnout symptoms and improving occupational well-being.

## Figures and Tables

**Table 1:** Descriptive statistics and Pearson’s *r* correlations among the study variables.

Construct	*N*	*Min*	*Max*	*M*	*SD*	1	2	3	4
1. Burnout—general	9705	8.00	64.00	40.17	8.94	-	0.93 ***	0.94 ***	0.56 ***
2. Burnout—Disengagement	9705	2.00	32.00	19.86	4.69	0.93 ***	-	0.74 ***	0.47 ***
3. Burnout—Exhaustion	9705	4.00	32.00	20.31	4.91	0.94 ***	0.74 ***	-	0.56 ***
4. Depression	9698	0.00	60.00	14.59	11.38	0.56 ***	0.47 ***	0.56 ***	-

*Note.* * *p* < 0.05, ** *p* < 0.01, *** *p* < 0.001.

**Table 2:** Model adequacy and goodness of fit indices of the basic models for burnout and depression.

Models	*Χ* ^2^	*df*	*P*	*Χ*^2^/*df*	RMSEA	PCLOSE	SRMR	IFI	AIC
Model—“Burnout”—two-factorial									
Model—“Burnout”— Simple CFA	12,379.19	103	<0.001	120.19	0.11	0.000	0.08	0.77	12,477.19
Model—“Burnout”— CFA with modification indices	7001.07	93	<0.001	75.28	0.09	0.000	0.07	0.87	7119.07
Model—“Burnout”—one-factorial									
Model—“Burnout”— Simple CFA	12,485.36	104	<0.001	120.05	0.11	0.000	0.08	0.77	12,581.53
Model—“Burnout”— CFA with modification indices	4243.00	86	<0.001	49.34	0.07	0.000	0.06	0.92	4375.00
Model—“Depression”									
Model—“Depression”— Simple CFA	13,514.71	170	<0.001	79.50	0.09	0.000	0.06	0.88	13,634.71
Model—“Depression”— CFA with modification indices	5663.03	161	<0.001	35.17	0.06	0.000	0.03	0.95	5801.03

*Note.* CFA = Confirmatory Factor Analysis; RMSEA = root mean square error of approximation; PCLOSE = *p* of Close Fit; SRMR = standardized root mean square residual; IFI = incremental fit index; AIC = Akaike information criterion. The respecifications of models were achieved based on error covariance modification indices.

**Table 3:** Model adequacy and goodness of fit indices of the combined models for burnout and depression.

Models	*Χ* ^2^	*df*	*P*	*Χ*^2^/*df*	RMSEA	PCLOSE	SRMR	IFI	AIC
Model 1									
Model 1—Simple CFA	52,365.66	594	<0.001	88.16	0.10	0.000	0.10	0.70	52,581.66
Model 1— CFA with modification indices	34,507.65	586	<0.001	58.89	0.08	0.000	0.09	0.80	34,739.65
Model 2									
Model 2—Simple CFA	29,460.04	591	<0.001	49.85	0.07	0.000	0.07	0.83	29,682.04
Model 2— CFA with modification indices	21,315.71	584	<0.001	36.50	0.06	0.000	0.07	0.88	21,551.71
Model 3									
Model 3—Simple CFA	29,638.35	593	<0.001	49.98	0.07	0.000	0.07	0.83	29,856.35
Model 3— CFA with modification indices	20,218.46	586	<0.001	34.50	0.06	0.000	0.07	0.89	20,450.46

*Note.* CFA = Confirmatory Factor Analysis; RMSEA = root mean square error of approximation; PCLOSE = *p* of Close Fit; SRMR = standardized root mean square residual; IFI = incremental fit index; AIC = Akaike information criterion. The respecifications of models were achieved based on error covariance modification indices.

## Data Availability

The data presented in this study are available on request from the corresponding author. The data are not publicly available due to ethical reasons.

## Appendix A

**Table A1:** Summary of items and factor loadings for Promax two-factor solution with PAF extraction for the OLBI scale.

Item	Factor Loading	
	1	2
	Factor 1: Reversed items 3 (α = 0.86)	
OLBI8r	0.81	0.00
OLBI4r	0.78	−0.07
OLBI3r	0.74	0.03
OLBI12r	0.70	0.06
OLBI9r	0.69	0.00
OLBI2r	0.66	−0.11
OLBI11r	0.51	0.10
OLBI6r	0.42	0.02
	Factor 2: Non-reversed items (α = 0.80)	
OLBI16	0.10	0.69
OLBI7	0.04	0.69
OLBI1	0.00	0.65
OLBI15	−0.08	0.64
OLBI14	−0.04	0.58
OLBI5	0.03	0.53
OLBI13	−0.07	0.42
OLBI10	0.13	0.41
% of variance	32.64%	7.97%

*Note.* The amount of total variance explained equals 40.60% and α = 0.88 for the entire measure.
